# Enhancing Employee Innovation Through Dependence on AI: The Mediating Role of Cognitive Flexibility and Moderating Effect of Job Complexity

**DOI:** 10.3390/bs16081274

**Published:** 2026-07-27

**Authors:** Zhiyong Han, Yanlong Zhang, Yonghong Zhu, Jingjing Liu

**Affiliations:** School of Business Administration, Anhui University of Finance and Economics, Bengbu 233030, China; hzyong@aufe.edu.cn (Z.H.); 3202400113@aufe.edu.cn (Y.Z.); 120081209@aufe.edu.cn (Y.Z.)

**Keywords:** cognitive flexibility, dependence on AI, innovative behavior, job complexity, Job Demands-Resources model

## Abstract

Drawing upon the Job Demands-Resources (JD-R) model, this study explores the mechanism through which Dependence on AI affects employees’ innovative behavior, focusing on the mediating role of cognitive flexibility and the moderating role of job complexity. Using questionnaire data from full-time employees collected between December 2024 and February 2025, this study conducts confirmatory factor analysis, correlation analysis, hierarchical regression and the Bootstrap test with AMOS, SPSS and the PROCESS macro. The results show that Dependence on AI significantly and positively predicts employees’ innovative behavior, and cognitive flexibility plays a partial mediating role between the two variables. Job Complexity serves as a negative moderator for the association between Cognitive Flexibility and Innovative Behaviors, and the positive linkage of Cognitive Flexibility to Innovative Behaviors becomes relatively weaker when Job Complexity is high. This study expands the cognitive mediating path through which Dependence on AI influences innovative behavior, refines the theoretical boundary of demand–resource interaction in the JD-R model, and provides practical implications for organizations to rationally guide human–machine collaboration and enhance employees’ innovative ability in intelligent scenarios.

## 1. Introduction

Generative AI reshapes the modes of knowledge work, and intelligent tools have emerged as carriers of human–machine collaboration capable of undertaking sophisticated cognitive tasks (Noy & Zhang, 2023; Yildirim-Erbasli et al., 2026). Extant research has uncovered the multifaceted impacts of AI on employee behaviors. Some studies demonstrate that AI empowers service innovation (Ahn et al., 2025), boosts well-being (C. Zhang et al., 2026; Hu et al., 2026), and elevates risk-taking propensity (Han et al., 2025). Conversely, other scholars argue that high perceived AI prevalence deteriorates employees’ work attitudes and exacerbates negative emotions (Brougham & Haar, 2018). At the team level, while AI streamlines decision-making efficiency, it tends to trigger role ambiguity and erode interpersonal trust (Carter & Wynne, 2024). This study centers on Dependence on Artificial Intelligence (DAI), a construct describing the extent of employees’ reliance on artificial intelligence for information retrieval and the completion of work duties (Brynjolfsson et al., 2018; Tang et al., 2023), and investigates its underlying mechanism shaping employee innovative behavior.

Dependence on AI represents a cognitive behavioral pattern, which is fundamentally distinct from constructs such as AI usage and AI trust. Dependence on AI is characterized by employees’ increased engagement with artificial intelligence technologies and applications for work assistance (Tang et al., 2023). AI usage generally denotes the objective frequency and scope with which employees deploy AI tools to complete job tasks at work (Tang et al., 2022). AI trust denotes positive attitudes and confidence stemming from the belief that AI possesses the capability to assist individuals in achieving specific objectives (Glikson & Woolley, 2020; D. Zhang et al., 2025). Automation dependence centers on automated systems as the core subject rather than artificial intelligence (Lee & Moray, 1992). AI literacy, by contrast, refers to individuals’ proficiency in understanding and practically operating AI-related technical concepts (J. Su et al., 2025). Extant literature predominantly holds negative perceptions of Dependence on AI, yet an increasing number of scholars have started to investigate its beneficial effects. For instance, Dependence on AI can boost work enthusiasm and stimulate innovative exploration (Zhao et al., 2025; Ye et al., 2025). Although existing research is abundant and diversified, few studies have unpacked the internal cognitive transmission pathways through which Dependence on AI shapes innovation.

To remedy the previously identified research lacunae, this study constructs a conceptual model grounded in the Job Demands–Resources (JD-R) framework (Bakker et al., 2005; Bakker & Demerouti, 2017) to unpack the underlying cognitive mechanisms via which Dependence on AI shapes employees’ innovative behavior. Cognitive flexibility refers to individuals’ capacity to adjust cognitive strategies, integrate diverse information, and generate creative solutions when confronted with novel situations (Nakhostin-Khayyat et al., 2024; X. Q. Wang et al., 2025). Cognitive psychology literature has validated it as a core determinant of innovative thinking (Nijstad et al., 2010; Zabelina & Robinson, 2010). Treating Dependence on AI as a job resource, the current study proposes that it exerts an impact on employee innovative behavior via a gain pathway of cognitive flexibility. Consistent with prior evidence, abundant job resources facilitate employees’ innovative conduct (H. Wang & Peng, 2022; Xia et al., 2026). Employees who rely on artificial intelligence at work exhibit enhanced capabilities in problem identification, information retrieval, and cognitive processing (Chen & Xiao, 2025; Eloundou et al., 2024), which constitute the core resources and critical prerequisites for engaging in innovative behavior.

Mainstream research on the JD-R model primarily focuses on the buffering effect of job resources against the depletion induced by job demands (Bakker et al., 2005), whereas a paucity of research has examined the moderating conditions under which high job demands reverse and suppress the gain effects of job resources. Job demands denote those work characteristics that entail physiological and psychological costs; managing heightened job demands necessitates that employees allocate considerable cognitive and behavioral resources (Bakker & Demerouti, 2017). Job design exerts pivotal impacts on a wide range of individual-, team-, and organizational-level outcomes (Morgeson & Campion, 2003). As a core job characteristic embedded in job design, job complexity (JC) captures the intensity of cognitive and information-processing demands inherent in tasks and is categorized as a type of job demand (Demerouti et al., 2001; Lewig et al., 2007). Shaw and Gupta (2004) defined job complexity as an integrative core psychosocial feature of jobs, which reflects the overall level of skill, cognitive, and mental investment required by work itself. Morgeson and Humphrey (2006) stated that job complexity represents the intricacy and execution difficulty of job tasks. Meanwhile, they highlighted that complex tasks compel employees to deploy extensive high-order skills, thereby generating greater cognitive load and challenge (Morgeson & Humphrey, 2006; Parker et al., 2021). Highly complex jobs consume the majority of employees’ cognitive and psychological resources, reducing the cognitive resources available for innovation and further impeding the transformation of cognitive advantages into innovative behavior. Accordingly, this study introduces job complexity as a moderator to examine whether such a suppression effect exists.

**Figure 1:**
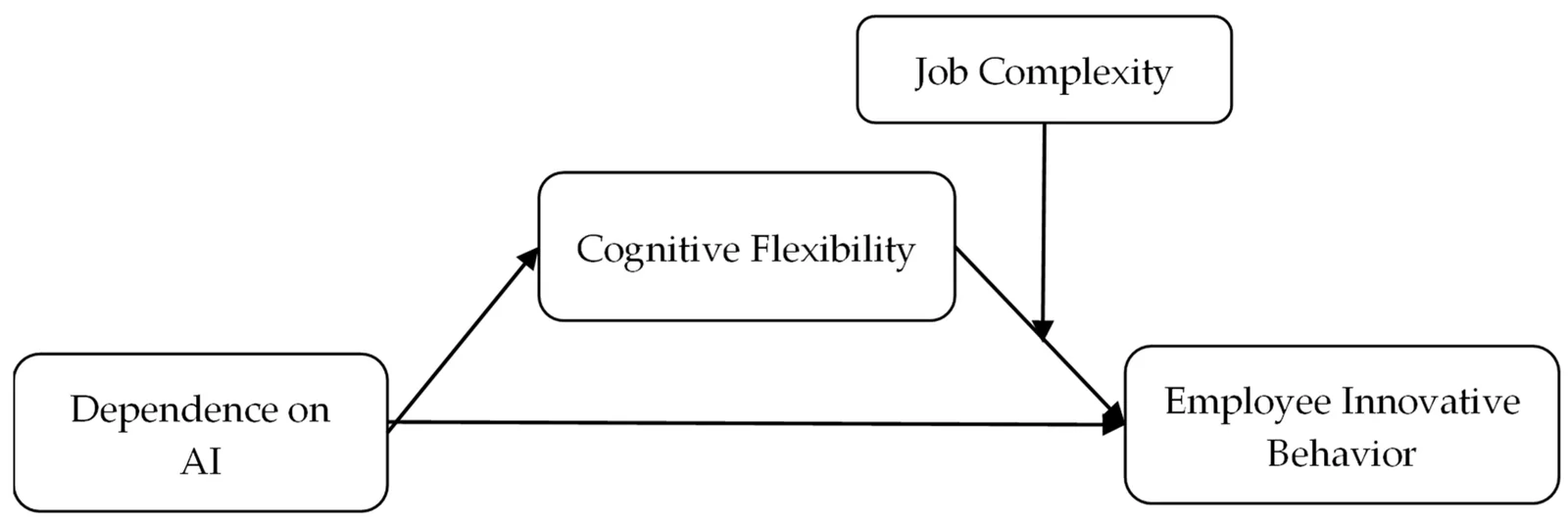
Theoretical Model.

This study adopts a questionnaire survey design to empirically verify the positive effect of Dependence on AI on employees’ innovative behavior via cognitive flexibility, as well as the moderating role of job complexity. Figure 1 illustrates the theoretical model proposed in this paper. The core contributions of the present research fall into three dimensions. First, this study takes Dependence on AI as the independent variable, which enriches the literature stream concerning artificial intelligence in the workplace. Second, cognitive flexibility is incorporated as the mediating variable to unpack the specific cognitive mechanism through which Dependence on AI shapes employee innovative behavior. Third, job complexity is introduced as a moderator to advance the theoretical development of the JD-R framework to a certain extent. Furthermore, the finding that highly complex jobs hinder the translation of cognitive resources into innovative behaviors partially corroborates the suppression effect of job demands on job resources.

## 2. Theoretical Foundation and Research Hypotheses

### 2.1. Job Demands-Resources Model

According to the JD-R theory, every work environment comprises two fundamental categories of elements: job demands and job resources. Job demands refer to job characteristics that require sustained physical or psychological exertion and thereby incur corresponding physical and psychological costs. In contrast, job resources represent workplace attributes that assist employees in accomplishing work objectives and facilitate personal growth and development (Demerouti et al., 2001; Lewig et al., 2007). The health-impairment path of job demands: persistently high job demands without adequate job resource support deplete employees’ energy, triggering burnout, work stress and physical and mental health issues. The motivational gain path of job resources: sufficient job resources satisfy employees’ fundamental psychological needs, boost work engagement, foster personal development, and ultimately yield superior job performance. Furthermore, the JD-R theory highlights the buffering function of resources against the adverse impacts of demands. Specifically, job resources can mitigate the detrimental effects of high job demands on employee well-being.

Extant JD-R theory categorizes job resources into four major types: organizational resources, social-interpersonal resources, personal resources, and technical job resources (Bakker & Demerouti, 2024; Xanthopoulou et al., 2009). Technical resources specifically refer to digital tools and intelligent systems that reduce cognitive load, expand individuals’ information boundaries, and assist in completing routine cognitive tasks. In terms of construct connotation matching, Dependence on AI fully aligns with the core attributes of technical job resources. The rationale is threefold: First, AI tools undertake repetitive cognitive labor such as information retrieval, data sorting, and standardized text generation, which directly lower employees’ basic job demands. Second, long-term reliance on AI interaction broadens employees’ information scope and diverse thinking perspectives to facilitate personal development. Third, during human–machine collaboration, AI provides references of multiple alternative solutions and delivers instrumental support for employees to achieve innovative work goals.

### 2.2. Dependence on AI and Employee Innovation

According to the JD-R framework, job resources refer to workplace attributes that facilitate employees’ attainment of work objectives (Bakker & Demerouti, 2024; Xanthopoulou et al., 2009). In digital work environments, artificial intelligence tools embody the typical characteristics of technical job resources. When employees rely on artificial intelligence for work, AI undertakes routine cognitive tasks including information retrieval, data processing, and pattern recognition, which directly reduces employees’ cognitive job demands. This enables employees to allocate supplementary energetic resources, time, and professional expertise to innovative behaviors that require in-depth exploration and novel ideation (Jia et al., 2024; Wilson & Daugherty, 2018). Notably, employees’ Dependence on AI primarily manifests as cognitive load reduction and supplementary information provision rather than comprehensive task substitution (Jiang & Peng, 2026; Yildirim-Erbasli et al., 2026), rendering the resource gain pathway more empirically explanatory. Accordingly, this study prioritizes theoretical analysis from the resource gain pathway perspective.

Dependence on AI provides robust algorithmic support for employees’ creative attempts and lowers their risk perception. Continuous monitoring and iterative optimization of workflows and outcomes powered by AI boost employees’ confidence and sense of security when exploring unfamiliar solutions or adopting novel approaches (Cannavale et al., 2022; Gandía et al., 2025). Such dependence mitigates the inherent uncertainty and failure costs embedded in the innovation process, emboldening employees to experiment with original ideas. This facilitates the translation of nascent concepts into actionable initiatives and ultimately enables the practical implementation of innovative outputs (Lehmann et al., 2026). Through human–AI interaction, employees can acquire, restructure and reconstruct work-related knowledge and experience (Alavi et al., 2024), which has been identified as a pivotal driver of creativity (H. Wang & Peng, 2022; Li et al., 2024). Building on the foregoing analysis, this study proposes:

**Hypothesis** **1.** *Dependence on AI positively affects employee innovation.*

### 2.3. Dependence on AI and Cognitive Flexibility

The JD-R theory contends that employees’ personal resources play a crucial role in shaping the link between job resources and later occupational outcomes (Bakker & Demerouti, 2017). Cognitive flexibility refers to an individual’s ability to flexibly adjust thinking patterns, perspectives and behaviors in response to changes in the external environment or internal goals (Nakhostin-Khayyat et al., 2024; X. Q. Wang et al., 2025), and is regarded as an important personal resource within the JD-R framework (Cui et al., 2026). In a study of professional writers, Noy and Zhang (2023) found that AI writing assistants not only improved writing efficiency but, more importantly, freed up authors’ cognitive resources for structural planning and creative ideation. Modern AI systems, especially large language models, can rapidly integrate massive information, identify complex patterns and generate diverse viewpoints, providing strong supplements to employees’ cognitive activities (Eloundou et al., 2024).

When employees depend on AI to process complex data, generate diverse solutions or predict future trends, they are encouraged to consider different possibilities, evaluate various options provided by AI, and integrate AI perspectives with their own professional knowledge. Such interaction with intelligent systems challenges and expands employees’ thinking boundaries, prompting them to develop stronger adaptability and multi-perspective thinking abilities (Huang et al., 2026). After AI systems undertake information gathering and preliminary analysis, employees can devote more cognitive resources to high-level cognitive activities that require cognitive flexibility, such as perspective shifting and creative combination (Cassenti et al., 2022; T. Ionescu, 2012). Therefore, as a kind of resource, AI not only provides convenience but also acts as a catalyst for the development of employees’ cognitive abilities, especially the improvement in cognitive flexibility. We propose:

**Hypothesis** **2.** *Dependence on AI exerts a positive effect on Cognitive Flexibility.*

### 2.4. The Mediating Role of Cognitive Flexibility

Innovative behavior is inherently a cognitively intensive activity that requires individuals to possess cognitive capacities such as flexibly integrating information, shifting mental frameworks, and exploring unconventional solutions (Anderson & Li, 2014; A. M. Ionescu et al., 2022; Mumford et al., 2023). Perry-Smith and Mannucci (2017) underscored the indispensability of cognitive flexibility for creative endeavors. Cognitive flexibility facilitates the integration and recombination of heterogeneous information (Z. W. Su et al., 2025).

Cognitive flexibility strengthens individuals’ tolerance for ambiguity and uncertainty. Individuals with high cognitive flexibility can endure temporary cognitive conflicts and ambiguous states, without prematurely fixating on a single solution. Instead, they are willing to explore and compare multiple alternatives (Zabelina & Robinson, 2010). X. Q. Wang et al. (2025) revealed that people with greater cognitive flexibility exhibit higher strategic variability. Such adaptability to uncertainty constitutes an essential psychological prerequisite for sustained innovation. Cognitive flexibility also improves individuals’ capacity for problem reframing. Many innovations stem not from direct resolution of original problems, but from redefining the problems themselves (McKay et al., 2024). Cognitive flexibility enables individuals to break free from established problem representation frameworks and thereby identify overlooked innovative opportunities. When individuals with high cognitive flexibility encounter obstacles amid innovative processes, they proactively adjust their mindsets and strategies, which ultimately yields superior individual innovative performance (M. N. Zhang et al., 2022).

Tang et al. (2022) illustrated that artificial intelligence takes over repetitive tasks with heavy cognitive load, freeing employees’ time to engage in higher-value activities that precisely demand cognitive flexibility and creativity (Tang et al., 2022). Zabelina and Robinson (2010) demonstrated that cognitive flexibility is significantly and positively linked to creative performance. Further empirical evidence from Wei et al. (2025) validated the predictive effect of cognitive flexibility on employees’ innovative behavior, confirming that cognitive flexibility exerts a positive influence on innovative performance. Integrating Hypothesis 1 and Hypothesis 2, we propose that:

**Hypothesis** **3.** *Cognitive flexibility plays a mediating role in the positive relationship between Dependence on AI and employee innovative behavior.*

### 2.5. The Moderating Role of Job Complexity

Within the JD-R framework, job complexity is inherently a high-load job demand that imposes multiple challenges on employees (Chung-Yan, 2010; Sung et al., 2017). This theoretical framework highlights that the interaction between job demands and personal resources jointly shapes work outcomes, and excessive job demands may lead to resource depletion (Tian et al., 2022; Baethge et al., 2019; Pan & Sun, 2018). Jia et al. (2024) contended that job complexity functions as a pivotal contextual determinant in human–AI interaction. Under conditions of high job complexity, employees confront greater pressure and elevated job demands, which in turn trigger intensified cognitive load (Prem et al., 2017; Xie & Johns, 1995) and attenuate the positive effects generated by job resources (Liu et al., 2026).

When job complexity is high, employees must allocate substantial cognitive resources to task analysis and procedural handling (C. J. Wang et al., 2014; Yang, 2018), leaving fewer disposable cognitive resources available for flexible deployment. Under such circumstances, even if employees possess high levels of cognitive flexibility, most of this cognitive capacity is occupied to cope with complex job tasks, with only a minor portion remaining to facilitate innovation transformation. By contrast, low-complexity jobs do not require sophisticated skills or diverse solution strategies (Chung-Yan, 2010) and consume limited cognitive resources, allowing employees to devote most cognitive resources to innovative conversion. In high-complexity work contexts, although cognitive flexibility still functions as a valuable personal resource, excessive job demands constrain its functional effectiveness. This leads to excessive depletion of psychological resources and heightened work stress, thereby restraining employees’ innovative capacity.

Meanwhile, drawing on the Componential Theory of Creativity proposed by Amabile (1988), the emergence of innovative behavior relies on the synergistic activation of three core elements: intrinsic motivation, creative thinking skills, and domain-relevant knowledge. Among these factors, intrinsic motivation exerts a decisive influence on individuals’ voluntary engagement in creative activities. Nevertheless, high job complexity triggers cognitive overload mechanisms, which significantly suppress employees’ intrinsic innovative motivation (Pan & Sun, 2018). Given the above theoretical elaboration, we develop the research hypotheses as follows:

**Hypothesis** **4.** *Job complexity plays a moderating role in the impact of cognitive flexibility on innovative behavior; that is, high job complexity inhibits the functioning of cognitive flexibility, thereby weakening the positive effect of cognitive flexibility on innovative behavior.*

## 3. Research Methods

### 3.1. Sample and Data Collection

This study conducted questionnaire surveys among full-time employees via the Credamo platform from December 2024 to February 2025, with a three-month data collection period. Data were collected at three separate time points. Specifically, demographic information, Dependence on AI and Job Complexity were measured at T1. Cognitive Flexibility was mainly assessed at T2 (six weeks after the first round of survey). Employees’ Innovative behaviors were investigated at Time 3 (another six weeks later).

To mitigate common method bias, several strategies were adopted in this study. First, anonymity and confidentiality were guaranteed to build respondents’ trust. Second, the items measuring the independent variable (Dependence on AI), mediator variable (Cognitive Flexibility), and dependent variable (Innovative behaviors) were scattered across different sections of the questionnaire to prevent the clustering of items from the same construct. Third, operational definitions were provided for abstract constructs. During data cleaning, invalid questionnaires were eliminated according to the following criteria: questionnaires with missing values accounting for 30% or above for a single variable, questionnaires with an overall missing rate of 20% or higher, responses with logical contradictions, and questionnaires featuring identical answers for 10 consecutive items or more. Ultimately, the final stage of data collection generated 405 valid questionnaires, corresponding to an effective response rate of 71.05%. For the feasibility and efficiency of data collection, convenience sampling was employed to recruit accessible full-time employees. However, the adopted sampling method may constrain the extent to which the study’s conclusions can be generalized to other contexts.

The sample of this study consists of data collected from 405 full-time employees. In terms of gender distribution, female respondents account for 67.4%, while male respondents account for 32.6%. With regard to age distribution, employees aged between 21 and 30 years make up 43.0%, and those aged 31 to 40 years account for 47.7%. In terms of educational background, respondents with college diploma or below take up 7.4%, bachelor’s degree holders account for 67.2%, and participants with master’s degree or above occupy 25.5%. In terms of working tenure, employees with no more than 5 years of work experience account for 35.8%, those with 6 to 10 years of working experience make up 44.0%, respondents with 11 to 15 years of working experience represent 12.8%, and only 7.4% of participants have over 16 years of work experience. From the perspective of industry distribution, the manufacturing sector accounts for 28.1%, and the internet industry accounts for 27.2%.

### 3.2. Measures of Variables

All measured variables in this study were assessed using well-established domestic and foreign scales that have been repeatedly adopted by numerous scholars in prior research. This study adopted the standard translation–back-translation procedure to convert English scales into Chinese, and made appropriate adjustments to the expressions according to research needs. All core research variables were measured using a 5-point Likert scale, where “1” indicated “strongly disagree” and “5” indicated “strongly agree”.

Dependence on AI (DAI, α = 0.781): Measured using the 3-item scale developed by Tang et al. (2023). A representative item is: “I depend on artificial intelligence to process or assist in work-related activities.”

Job Complexity (JC, α = 0.821): Measured using the 3-item scale developed by Shaw and Gupta (2004). A representative item is: “My job is very complex.”

Cognitive Flexibility (CFI, α = 0.897; Cronbach’s α coefficients of the two subscales were 0.845 and 0.810, respectively). This variable was measured using the Cognitive Flexibility Inventory originally developed by Dennis and Vander Wal (2010). The scale was later translated into Chinese by domestic scholars and verified to have satisfactory reliability and validity in the Chinese cultural context (Y. Wang et al., 2016), consisting of 20 items in total. A representative item is “I am good at handling various situations flexibly.” Following the recommendations proposed by Hair et al. (2010), Items 6, 9 and 15 were removed from the formal analysis due to their low factor loadings in this study.

Innovative Behavior (IB, α = 0.759): Measured using the 6-item scale developed by Scott and Bruce (1994). A representative item is: “I generate ideas for exploring new technologies, processes, methods and products.”

Control Variables: To avoid confounding the research results, gender, age, work experience and education level were set as control variables in this study.

### 3.3. Analysis Strategy

First, this study used AMOS 24.0 to conduct confirmatory factor analysis (CFA) to examine the discriminant validity of the four core variables: Dependence on AI, cognitive flexibility, job complexity, and innovative behavior. Second, SPSS Statistics 27.0 was used to calculate the mean and standard deviation of each variable, as well as to perform bivariate correlation analysis and regression analysis. Finally, the PROCESS 4.1 macro in SPSS was employed to test the mediating effect and moderating effect.

## 4. Results

### 4.1. Confirmatory Factor Analysis

To test the discriminant validity of the core variables, this study conducted confirmatory factor analysis (CFA), and the results are presented in Table 1. The results show that the four-factor model including Dependence on AI, cognitive flexibility, job complexity, and innovative behavior fitted the data well (χ^2^/df = 1.899, RMSEA = 0.047, CFI = 0.915, IFI = 0.915, SRMR = 0.048). The above results verify that the core variables have satisfactory discriminant validity.

Among the measured variables, the abbreviated scales of Dependence on AI (DAI) and Job Complexity (JC) yielded average variance extracted (AVE) values of 0.56 and 0.60, and composite reliability (CR) values of 0.85 and 0.87 respectively, which met the recommended evaluation criteria. Although the AVE values of Cognitive Flexibility (CFI) and Innovative behaviors (IB) were slightly below the conventional threshold, their CR coefficients both exceeded 0.85, accompanied by favorable model fit indices. Accordingly, the convergent validity of these two constructs was deemed acceptable (Lam, 2012).

### 4.2. Common Method Bias Test

This study adopted a multi-wave data collection strategy to reduce the potential impact of common method bias. First, Harman’s one-factor test (Podsakoff et al., 2003) was used to conduct factor analysis on all observed items of the four variables: Dependence on AI, cognitive flexibility, job complexity, and innovative behavior. The results showed that multiple common factors were extracted, and the variance explanation rate of the first common factor was only 29.4%, which was below the empirical threshold. In addition, this study further conducted a confirmatory factor analysis with an added common method factor for verification, that is, a latent method factor was incorporated into the CFA model (Gu & Wen, 2017). The results showed that the model fit indices were not significantly improved: the changes in RMSEA and SRMR were both less than 0.05, and the changes in CFI and IFI were both less than 0.01. This indicates that there is no severe common method bias in the sample data, and subsequent statistical tests can be carried out.

### 4.3. Correlation Analysis

Table 2 presents the means, standard deviations, and correlation coefficients of the control variables, Dependence on AI (DAI), job complexity (JC), cognitive flexibility (CFI), and innovative behavior (IB). As shown in the table, innovative behavior (IB) is significantly and positively correlated with cognitive flexibility (CFI), job complexity (JC), and Dependence on AI (DAI). The positive correlation between DAI and CFI is also significant. The meaningful relationships identified among the variables provide initial empirical support for all the hypotheses advanced in this study.

### 4.4. Hypothesis Testing

This study used hierarchical regression analysis to test the research hypotheses, and the results of hierarchical regression are shown in Table 3. The potential multicollinearity of all predictive variables was examined via the variance inflation factor (VIF). The results showed that the maximum VIF value was 3.48, far below the common threshold of 5, indicating that multicollinearity would not significantly affect the regression equation.

Hypotheses 1 and 2 were tested using SPSS, and the results are presented in Table 3. Hypothesis 1 proposes that DAI has a positive effect on IB. As shown in Model 4 in Table 3, DAI has a significantly positive effect on IB (b = 0.541, *p* < 0.001), thus Hypothesis 1 is supported. Hypothesis 2 proposes that DAI has a positive effect on CFI. According to Model 2 in Table 3, DAI has a significantly positive effect on CFI (b = 0.409, *p* < 0.001), so Hypothesis 2 is supported. Further analysis reveals that CFI has a significantly positive effect on IB (Model 6: b = 0.614, *p* < 0.001). After incorporating CFI into the model (Model 5), the coefficient of DAI decreases to 0.351 (*p* < 0.001), indicating that CFI plays a mediating role between DAI and IB. Furthermore, to test the robustness of the mediating effect of CFI, the PROCESS macro embedded in SPSS was adopted with the Bootstrap method based on 5000 resamples. The results revealed that the mediating effect value was 0.105, and the 95% confidence interval (95% CI = [0.066, 0.155]) excluded zero (see Table 4). This finding verifies that Cognitive Flexibility exerts a significant mediating effect between DAI and IB, thus Hypothesis 3 is supported.

**Figure 2:**
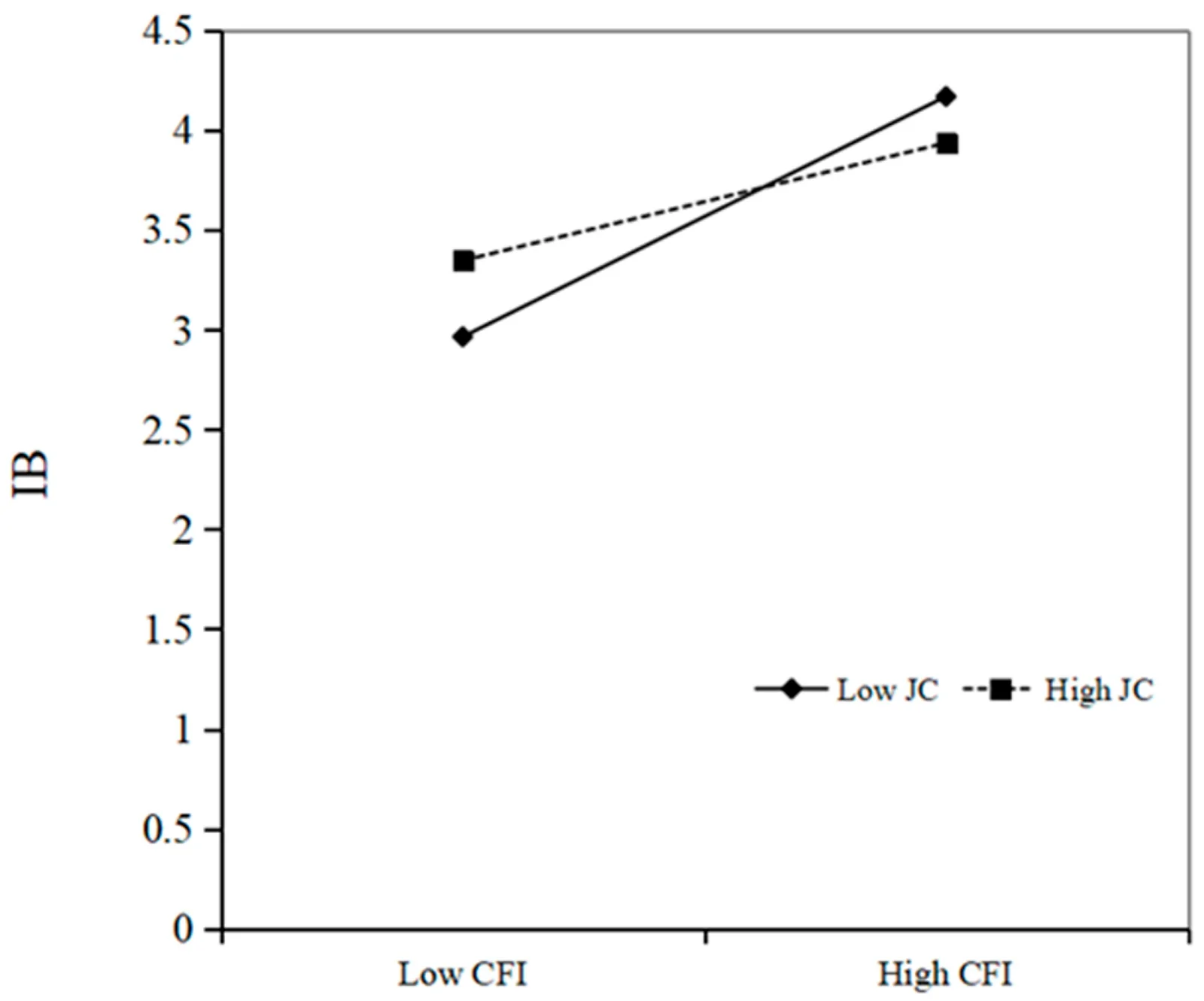
Moderating Role of Job Complexity.

The results of Model 8 show that the interaction term between CFI and JC has a significant effect on IB (b = −0.258, *p* < 0.001). Simple slope analysis (see Figure 2) indicates that CFI has a positive effect on IB under both high- and low-JC conditions. However, the slope is significantly smaller under high-JC conditions, suggesting that JC moderates the effect of CFI on IB and weakens this positive relationship. Hypothesis 4 is supported. To enhance the reliability of the empirical results, Model 1 in the SPSS PROCESS macro was utilized with 5000 Bootstrap resamples to re-examine the moderating effect. The results demonstrated a significant moderating effect (b = −0.41, 95% CI = [−0.5665, −0.2137]). Meanwhile, we performed reverse causality tests on the moderation effect. The insignificant findings provide partial evidence supporting the plausibility of our model to a certain degree. Accordingly, CFI shows a positive association with IB across both high and low Job Complexity contexts. Nevertheless, the magnitude of this positive linkage is relatively weaker under high Job Complexity compared with low Job Complexity settings. This observation suggests that lower levels of Job Complexity may facilitate the more efficient conversion of cognitive resources into innovative outcomes.

## 5. Discussion

Drawing on the JD-R model, this study explores the internal mechanism through which Dependence on AI affects employees’ innovative behavior via cognitive flexibility, as well as the contextual constraining effect of job complexity within this process. The empirical results reveal that DAI positively predicts employee innovative behavior, with cognitive flexibility serving as a partial mediator. Furthermore, job complexity negatively moderates the facilitating effect of cognitive flexibility on innovative behavior; high job complexity reduces the efficiency of converting cognitive resources into innovative actions. These findings outline a complete theoretical chain: as a type of technical job resource, Dependence on AI activates the gain pathway of cognitive flexibility by broadening employees’ information scope and diversifying their thinking perspectives. Nevertheless, the effectiveness of translating such resources into innovative behavior is not universal. When JC reaches a high level, the surplus stock of cognitive resources is occupied, making it difficult for flexible cognition to translate into tangible innovative actions. This evidence supports the core proposition of the JD-R model that job resources shape work outcomes through the gain pathway (Bakker & Demerouti, 2017). Meanwhile, it substantially refines the interactive boundary between job demands and job resources embedded in this theory: job demands are not merely factors buffered by resources, but may conversely constrain the functional utility of job resources.

Another worthy research question emerges: if job complexity limits the effective utilization of job resources, can it also disrupt the conversion of AI-generated resources into cognitive resources? Supplementary analyses in this study tested this first-stage moderating effect. The results revealed marginally significant moderation with a small effect size and broad confidence intervals, whose robustness was substantially lower than that of the second-stage moderating effect. This implies that job complexity exerts mild yet limited interference at the resource input stage. Grounded in the JD-R framework, this research focuses on the transformation from cognitive resources to behavioral outcomes, specifically how job complexity depletes executive resources and hinders innovation implementation. The conditions under which job complexity weakens the pathway from AI resources to cognitive resources lie outside this study’s theoretical scope. The supplementary findings above only deliver preliminary empirical clues, which await verification via systematic theoretical reasoning and rigorous research designs in future work.

### 5.1. Theoretical Implications

First, this study contributes to the literature on Dependence on AI. Most existing AI-related research is grounded in “AI usage”. For instance, Budhwar et al. (2023) and Mira et al. (2022) pointed out that the automated content generation capability of AI significantly improves the efficiency and quality of advertising copy creation and emerging market identification, suggesting that using AI can make work life simpler and better. However, research on artificial intelligence should not be limited to a single dimension. Therefore, this study explores the issue from the perspective of Dependence on AI, contributing to multi-perspective research on artificial intelligence.

Second, this study provides a modest incremental contribution to the research on the mediating mechanism through which Dependence on AI influences employees’ Innovative behaviors. Existing studies have taken creative process engagement and information overload as mediating variables and explained how AI dependence affects innovation from the perspectives of input and information (Cui et al., 2026), yet insufficient attention has been paid to cognitive mechanisms. This study regards cognitive flexibility as the core mediating variable and verifies the transmission path that Dependence on AI promotes employee innovative behavior by improving their cognitive flexibility, identifying cognitive flexibility as a key cognitive link between Dependence on AI and innovative behavior. It makes a modest incremental contribution to the mechanism whereby Dependence on AI affects Innovative behaviors from the cognitive perspective.

Third, this study refines the theoretical boundary of demand–resource interaction within the JD-R model, and verifies the inhibitory effect of job demands on the effectiveness of job resources to a certain extent. The JD-R theory highlights that job resources can buffer the adverse impacts triggered by job demands, namely job resources alleviate the negative effects of high job demands on employee well-being. Existing scholarship has mainly investigated the cushioning function of job resources against elevated job demands (Cui et al., 2026; Li et al., 2025; Lesener et al., 2019), while scant research has explored whether job demands undermine the positive effects of job resources. Regarding Job Complexity as a core job demand, this study finds that it weakens the positive influence of Cognitive Flexibility on Innovative behaviors. That is, high Job Complexity inhibits the functional effectiveness of CFI as a personal job resource. This finding supplements the interaction effect theory of the JD-R model, reveals that the effectiveness of technological job resources is not universal but constrained by contextual characteristics of job demands, and provides new empirical evidence for understanding the interactive relationship between job demands and job resources in intelligent workplace scenarios.

### 5.2. Practical Implications

First, guide employees to maintain moderate Dependence on AI. Organizations should clarify applicable AI scenarios via training, and encourage staff to actively utilize AI for tasks such as information collection, data analysis and routine solution generation to expand their thinking boundaries, so that AI can practically function as a technical job resource to boost cognitive flexibility.

Second, prioritize the cultivation of cognitive flexibility. As a critical mediator translating Dependence on AI into innovative behavior, cognitive flexibility should be integrated into the competency development system. Training activities including cross-domain case analysis, multi-scheme design and cross-departmental collaboration can motivate employees to proactively improve their cognitive flexibility and fully capitalize on cognitive benefits brought by AI.

Third, conduct differentiated management of job complexity to mitigate contextual inhibitory effects. For high-complexity positions (e.g., R&D and strategic posts), organizations shall reasonably regulate the degree of Dependence on AI and establish supporting mechanisms of professional consultation and problem seminars to ease cognitive load. For low-complexity positions (e.g., basic data processing and routine document drafting), appropriately higher Dependence on AI should be advocated to cut cognitive costs and stimulate process innovation.

### 5.3. Limitations and Future Research

Although data were collected at multiple time points in this study, all variables were measured via employees’ self-reported surveys, which may lead to common method bias (Podsakoff et al., 2016) and social desirability bias. Future research can verify these findings using multi-source data, including supervisor evaluations, coworker feedback and objective indicators. In addition, several scales have uneven item coverage with marginally acceptable reliability; follow-up studies need to optimize measurement instruments.

Second, this study only investigates the moderating effect of job complexity and ignores other boundary conditions at the individual level (e.g., innovative personality) and organizational level (e.g., organizational culture). Future scholars can construct multi-layer moderating models. Moreover, complex work tasks may generate facilitating effects and even drive employees’ competence development, yet such effects vary across personality traits, which can be a promising direction for subsequent research.

Finally, this study is situated within the Chinese context, so the generalizability of its conclusions needs cross-industry and cross-cultural validation. Rapid AI technological iteration reshapes human–machine collaboration modes and may reduce the temporal validity of the findings. Future longitudinal research can track the long-term evolutionary trajectories of Dependence on AI on individual cognition and behaviors, while updating measurement instruments to keep pace with technological progress.

## 6. Conclusions

Based on the JD-R model, this study systematically examines how Dependence on AI (DAI) positively influences innovative behavior (IB) through cognitive flexibility (CFI), as well as the moderating role of job complexity (JC) in this process, using empirical evidence. The findings not only provide important theoretical implications for understanding the cognitive mechanisms of human–machine collaboration in the digital era, but also offer empirical evidence for organizations to implement human resource management practices in the AI era.

## Figures and Tables

**Table 1:** Results of confirmatory factor analysis.

Model	χ^2^	df	χ^2^/df	CFI	IFI	RMSEA	RMR	SRMR
Four-Factor Model (DAI, CFI, JC, IB)	704.452	371	1.899	0.915	0.915	0.047	0.023	0.048
Three-Factor Mode (DAI, CFI + JC, IB)	1158.457	374	3.097	0.799	0.801	0.072	0.053	0.069
Three-Factor Mode (DAI + CFI, JC, IB)	992.164	374	2.653	0.842	0.843	0.064	0.036	0.0604
Three-Factor Mode (DAI, CFI, JC + IB)	1146.901	374	3.067	0.802	0.804	0.072	0.053	0.0685
Three-Factor Mode (DAI + JC, CFI, IB)	1149.27	374	3.073	0.802	0.803	0.072	0.053	0.0681
Two-Factor Mode (DAI + CFI + JC, IB)	1444.156	376	3.841	0.727	0.729	0.06	0.084	0.0778
Two-Factor Mode (DAI + IB + JC, CFI)	1265.145	376	3.365	0.773	0.774	0.077	0.054	0.0723
One-Factor Mode (DAI + CFI + JC + IB)	1537.209	377	4.077	0.703	0.705	0.087	0.06	0.0795

Note: DAI = Dependence on AI, CFI = Cognitive Flexibility, JC = Job Complexity, IB = Innovative behaviors.

**Table 2:** Means, Standard Deviations, and Correlation Coefficients of Variables (N = 405).

Variable	1	2	3	4	5	6	7	8	9	10
1. Gender										
2. Age	0.119 *									
3. edu	−0.002	0.084								
4. work	0.084	0.826 **	−0.033							
5. position	0.08	0.487 **	0.263 **	0.442 **						
6. section	0.038	0.066	−0.178 **	0.159 **	−0.081					
7. DAI	−0.029	0.056	0.093	0.014	0.121 *	−0.226 **				
8. JC	−0.058	0.041	0.04	0.089	0.051	−0.107 *	0.061			
9. CFI	−0.039	0.163 **	0.133 **	0.110 *	0.210 **	−0.185 **	0.451 **	0.041		
10. IB	−0.026	0.079	0.106 *	0.058	0.191 **	−0.133 **	0.557 **	0.104 *	0.625 **	
Mean	1.6741	2.6543	2.1951	1.9728	2.0173	3.5531	3.7457	3.6765	4.2154	4.2428
SD	0.4693	0.72372	0.57933	1.02771	0.93724	2.24276	0.78195	0.84621	0.3538	0.43042

Note: * *p* < 0.05, ** *p* < 0.01; DAI = Dependence on AI, CFI = Cognitive Flexibility, JC = Job Complexity, IB = Innovative behaviors.

**Table 3:** Regression Analysis Results for Hypothesis Testing.

Variables	CFI		IB					
	Model 1	Model 2	Model 3	Model 4	Model 5	Model 6	Model 7	Model 8
gender	−0.057	−0.044	−0.036	−0.019	−0.003	−0.001	0.003	0.024
age	0.132	0.108	0.002	−0.029	−0.076	−0.079	−0.068	−0.033
edu	0.057	0.046	0.04	0.026	0	0.006	0.003	−0.015
work	−0.025	−0.01	0.002	0.021	0.018	0.017	−0.001	−0.018
position	0.133	0.098	0.172 **	0.126 *	0.086 *	0.091	0.091	0.075
section	−0.167	−0.08	−0.111 *	0.003	0.037	−0.009	0.001	0.038
DAI		0.409 ***		0.541 ***	0.351 ***			
CFI					0.466 ***	0.614 ***	0.613 ***	0.625 ***
JC							0.077 *	0.181 ***
CFI×JC								−0.258 ***
R2	0.087	0.244	0.053	0.327	0.492	0.397	0.403	0.457
DR2	0.087	0.157	0.053	0.274	0.165	0.344	0.006	0.054
F	6.343	18.308	3.733	27.546	47.993	37.406	33.438	36.973

Note: * *p* < 0.05, ** *p* < 0.01, *** *p* < 0.001.

**Table 4:** Decomposition of Total Effects.

Effect Type	Effect	SE	95% CI		Relative Effect Size
Total Effect	0.298	0.023	0.252	0.344	
Direct Effect	0.192	0.022	0.148	0.236	64.60%
Indirect Effect	0.105	0.023	0.066	0.155	35.40%

## Data Availability

The data presented in this study are available on request from the corresponding author.
